# Vitamin D-induced vitamin D receptor expression induces tamoxifen sensitivity in MCF-7 stem cells via suppression of Wnt/β-catenin signaling

**DOI:** 10.1042/BSR20180595

**Published:** 2018-12-07

**Authors:** Wei Zheng, Bofeng Duan, Qian Zhang, Linna Ouyang, Wei Peng, Fuyong Qian, Yibin Wang, Shiting Huang

**Affiliations:** 1 Department of Breast and Thyroid Surgery, The Third People’s Hospital of Shenzhen, Shenzhen, Guangdong 518112, China; 2 Teaching and Research Section of Surgery, Xiangnan University Affiliated Hospital, Chenzhou, Hunan 423000, China

**Keywords:** Breast cancers, Cancer stem cells, Chemotherapy resistance, Tamoxifen, Vitamin D receptor

## Abstract

**Objective:** Cancer stem cells (CSCs) are responsible for the drug resistance of breast cancers. Vitamin D deficiency promotes tumor resistance. The present study examined the effect of vitamin D and vitamin D receptor (VDR) expression on the tamoxifen resistance of CSCs. **Methods:** MCF-7 cells were treated with 1,25(OH)_2_D_3_ and their levels of VDR expression, viability, and apoptosis were detected. CD133^+^ MCF-7 stem cells were identified and transfected with a VDR-overexpression plasmid. The tamoxifen concentration that reduced MCF-7 cell viability by 50% (IC_50_) was determined. The activation of Wnt/β-catenin signaling was also investigated. **Results:** Vitamin D reduced the viability of MCF-7 cells and promoted their apoptosis. Vitamin D enhanced VDR expression and induced DNA damage. When CD133^+^ stem cells were separated from MCF-7 cells, the IC_50_ of tamoxifen for stem cells was significantly higher than that of parental MCF-7 cells, suggesting a higher tamoxifen resistance in MCF-7 stem cells. Levels of VDR expression and Wnt/β-catenin signaling in CD133^+^ cells were markedly lower and higher than those in CD133^−^ cells, respectively. Stem cells transfected with VDR overexpression plasmids showed decreased tamoxifen IC_50_ values, viability, spheroid formation, and expression of Wnt and β-catenin proteins when compared with control cells. Cell apoptosis was increased by transfection with a VDR overexpression plasmid. Finally, the inhibitory effects induced by VDR overexpression could be reversed by the VDR inhibitor, calcifediol. **Conclusion:** Stem cells contributed to the tamoxifen resistance of MCF-7 cells. Vitamin D-induced VDR expression increased the sensitivity of MCF-7 stem cells to tamoxifen by inhibiting Wnt/β-catenin signaling.

## Introduction

Breast cancer is the second most common malignancy in women, and accounts for almost 30% of new cancers and 15% of cancer-related deaths [1,2]. Breast cancer is an malignancy with a poor prognosis because of its genetic and phenotypic diversity, delayed diagnosis, and resistance to radiation and chemotherapy [3].

Cancer stem cells (CSCs) comprise a small number of cells within tumors and have features similar to normal stem cells, such as embryonic stem cells [4,5]. It has been well documented that CSCs are responsible for the initiation, metastasis, and drug resistance of cancer cells [6–8]. CSCs from malignancies have been identified and reported to contribute to tumor metastasis and chemoradiotherapy resistance [9,10]. It is reported that breast cancer is driven by breast CSCs, which in turn drive tumor formation, metastasis, and resistance to chemotherapy [9–11]. The persistent activation of some conserved signaling pathways in CSCs, including the Notch and Wnt signaling pathways, guarantees the continued development of breast tumors and their metastasis [5,12]. High levels of CSCs in breast cancer specimens are always predictive of metastasis, resistance to chemotherapeutic agents, and a poor clinical outcome [4,13,14].

Vitamin D deficiency is a common factor found in patients with many disorders, including breast cancer, and a low vitamin D level is advantageous for tumor development, drug resistance, and metastasis [1,15,16]. Women with sufficient supplementation of calcium and vitamin D showed a decreased risk for invasive breast cancers [17].

Vitamin D is not only a vitamin, but also a multifunctional pro-hormone and a precursor to calcitriol [1,25-dihydroxy-vitamin D3 (1,25(OH)_2_D_3_)], which is a potent steroid hormone [18]. The etiology of vitamin D deficiency-mediated cancers is characterized by an alteration in the oncogenic Wnt/β-catenin signaling pathway or the APC/β-catenin/TCF tumor suppressor pathway [5,19,20]. So et al. [21] found that administration of 1,25(OH)_2_D_3_ or its analogs decreased Notch ligand levels, inhibited Notch 1 signaling, and reduced the CSC numbers among breast cancer cells. There is increasing evidence showing that vitamin D may exert therapeutic effects in breast cancer patients by inhibiting certain signaling pathways found in CSCs. Moreover, the resistance of breast cancer to tamoxifen is mediated by CSCs [22]. However, less information is known about the inhibitory effect of vitamin D on breast cancer, and the roles played by the vitamin D receptor (VDR) and its analogs in the resistance of breast CSCs to tamoxifen.

The present study explored the effect of vitamin D on breast cancer cells, including their viability, apoptosis, and VDR expression. The influence of VDR expression on the tamoxifen resistance of MCF-7 stem cells will be discussed. The association between Wnt/β-catenin signaling and VDR-mediated tamoxifen resistance in MCF-7 stem cells was studied. Our study results provide new insights into the association of tamoxifen resistance in MCF-7 stem cells with VDR expression and Wnt/β-catenin signaling.

## Materials and methods

### Cell culture and vitamin D treatment

Human MCF-7 breast cancer cells were purchased from ATCC (Manassas, VA, U.S.A.). The cells were maintained and expanded in DMEM (Hyclone, Logan, UT, U.S.A.) supplemented with 10% FBS (Sigma-Aldrich, St. Louis, MO, U.S.A.) at 37°C, in a 5% CO_2_ atmosphere. Cells were treated with 0, 0.001, and 0.1 µM 1,25(OH)_2_D_3_ at 37°C with 5% CO_2_. Each cellular treatment was performed in triplicate. The VDR inhibitor calcifediol (S1469) was used at a concentration of 0.1 μM to block the VDR.

### Fluorescence-activated cell sorting and CD133^+^ CSC treatment

CD133^+^ stem cells were separated from MCF-7 cells by use of fluorescence-activated cell sorting methods [23]. In brief, cells were centrifuged and labeled with a FITC-conjugated CD133 antibody (eBiosciences, San Diego, CA, U.S.A.) for 1 h; after which, they were sorted using a Becton Dickinson (BD) fluorescence-activated cell sorter (BD Biosciences, San Jose, CA, U.S.A.). Individual CD133^+^ stem cells were manually removed under a microscope and maintained in serum-free DMEM/F-12 medium (Hyclone) that was supplemented with 2% B27 (Invitrogen, Life Technologies, Grand Island, NY, U.S.A.), 0.4% BSA (Sigma-Aldrich), 1% penicillin/streptomycin, bFGF, and EGF (20 ng/mL each, PeproTech, Inc., Rocky Hill, NJ, USA), insulin (10 µg/ml, Sigma-Aldrich), hydrocortisone (0.5 µg/ml, Invitrogen), and sodium glutamate (2 mM, Invitrogen) at 37°C and 5% CO_2_ [24].

### VDR expression plasmid construction and CSC transfection

The VDR-overexpression plasmid was constructed by cloning the full length VDR CDS sequence into a pcDNA3.0 expression vector (GeneChem Co. Ltd., Shanghai, China). The full length VDR gene *CDS* was obtained from MCF-7 cells by using the VDR forward primer (5′-GGGGTACCATGGAGGCAATGGCGGC-3′) and reverse primer (5′-CCGCTCGAGTCAGGAGATCTCATTGCCAAACA-3′). The PCR products and pcDNA3.0 vectors were digested with KpnI (Thermo Fisher Scientific, Waltham, MA, U.S.A.) and XhoI (Thermo Fisher Scientific) at 37°C for 2 h, and then ligated by incubation with T4 DNA Ligase (Thermo Fisher Scientific) at 22°C for 2 h. The purified products (0.4 µg of plasmids) and empty vectors (negative controls) were used for transfection into CSCs (100 μl, 1 × 10^5^/ml). The transfections were performed using 0.5 µl of Lipofectamine™ 2000 (Invitrogen) according to the manufacturer’s instructions. Cells were incubated in serum-free DMEM/F-12 (Hyclone) medium that was supplemented with the factors described above at 37°C with 5% CO_2_ and then harvested at 48 h after cell transfection. All experiments were performed in triplicate.

### Spheroid SEM imaging

An ultrastructure analysis of CSC spheroids was performed using SEM imaging. Aliquots of isolated single cells (5 × 10^4^) were seeded into 24-well agar-coated plates and cultured under the conditions described above for 7 days. After being treated with methods recently described by Boo et al. [24].

### Soft agar colony formation assay

Soft agar colony formation was performed as describe [24]. Briefly, a mixture, consisted by 1.2% agar solution, 2× DMEM medium and about 2000 cells, was immitted into 96-well plate, and then plates were transfer into incubator for 10 days. Finally, plates were observed and photographed under microscope.

### Immunofluorescence assay

Spheres derived from CD133^+^ CSCs were centrifuged on slides by using cytospins according to recently described methods [6]. Next, cells were fixed and prepared with 0.1% Triton, blocked with BSA, and then incubated in solutions that contained FITC-conjugated CD33 antibody (1:500, eBiosciences); after which, they were incubated with an IgG-FITC fluorescent antibody (1:500, eBiosciences) and stained with DAPI. For DNA damage determination, Hoechst 33258 staining solution (Beyotime Institute of Biotechnology, Shanghai, China) was added into solutions that contained fixed cells and incubated for 30 min at room temperature. The cells were then examined under an Olympus confocal microscope (FV 1000, Olympus, Tokyo, Japan).

### Drug sensitivity assay

The sensitivity of parental MCF-7 cells and separated CSCs (CD133^+^) to the chemotherapeutic drug tamoxifen was measured using the MTT assay (Sigma-Aldrich) [24]. MCF-7 cells and CD133^+^ CSC spheroids were placed into 96-well plates at the conditions described above. Spheroids in 3D format and 2D format, and monolayer cells were dissociated into single cells, filtered, and counted. Next, aliquots containing 5 × 10^5^ cells were placed into the wells of culture plates that were supplemented with tamoxifen (0–32 µM) and incubated for 96 h. MTT solution was then added to each well (20 µl/well) and the cells were incubated for another 4 h prior to addition of DMSO (170 µl/well). The absorbance of cell cultures was detected using a microplate reader (Bio-Rad, Hercules, CA, U.S.A.). The percentage of viable cells and degree of cell cytotoxicity were determined by comparing the absorbance of treated cells with that of control cells (defined as 100%). Dose–response curves for the MCF-7 cells and CSCs were constructed, and the tamoxifen concentration that produced a 50% reduction in cell viability (IC_50_) was calculated.

### Flow cytometric analysis

For apoptosis analysis, single cells were seeded into the wells of 6-well plates (5 × 10^5^ cells/well) and incubated for 24 h; after which, 1,25(OH)_2_D_3_ was added and the cells were incubated for an additional 48 h. Next, the cells were harvested, digested to single cells using 0.25% trypsin (Gen-View Scientific, Inc., El Monte, FL, U.S.A.), and prepared for apoptosis detection that was performed after adding 200 µl of Annexin V/Propidium Iodide (PI) staining solution (BD Biosciences). For the EdU proliferation assay, cells in 96-well plates were first treated with 1,25(OH)_2_D_3_ for 48 h and then treated with 50 μM EdU solution for 2 h (100 μl/well, RiboBio, Guangzhou, Guangdong, China). The cells were then analyzed by flow cytometry as described in the manufacturer’s instructions. All flow cytometric analyses were performed using a BD FACS Calibur™ flow cytometry (BD Biosciences).

### Western blot analysis

Total cellular proteins were isolated from MCF-7 cells and CSC spheroids using lysis buffer (Beyotime). Samples of protein (35 µg) were separated by 10% SDS/PAGE (Sangon, Shanghai, China), and the separated protein bands were transferred onto PVDF membranes (Millipore, Whatman, Germany), which were then blocked with 5% skimmed milk (Beyotime). Next, the membranes were incubated with specific primary monoclonal antibodies against VDR (1:400, Abcam, Cambridge, U.K.), Wnt (1:1000, Abcam), β-catenin (1:5000, Abcam), and GAPDH (1:10,000, Abcam) at room temperature for 1 h. HRP Goat anti-Rabbit/mouse IgG (1:20,000, Boster Biotechnology Ltd., Wuhan, China) was used as the secondary antibody. The membranes were developed with an ECL system (Millipore, Billerica, MA, U.S.A.), and Image-Pro Plus 6.0 software (Media Cybernetics, Inc., Bethesda, MD, U.S.A.) was used to determine the optical density of each protein blot.

### Statistical analysis

All statistical analyses were performed using GraphPad Prism 6.0 software, and results are shown as the mean ± S.D. Student’s *t* test was used to analyze differences, and *P*-values <0.05 were considered statistically significant.

## Results

### Vitamin D treatment suppressed cell proliferation and induced cell apoptosis

**Figure 1:**
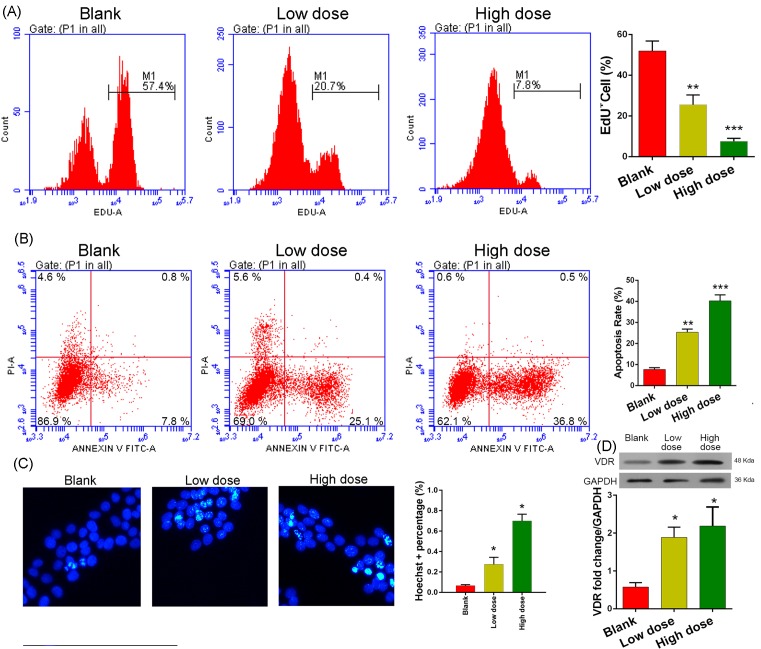
Effect of vitamin D on cell proliferation and apoptosis (**A**) EdU flow cytometric analysis. (**B**) Cell apoptosis analysis by flow cytometry. (**C**) Hoechst 33258 immunofluorescence assay; ×400 magnification. (**D**) Expression of the VDR protein in cells as determined by Western blot analysis. GAPDH was used as reference control. MCF-7 cells were treated with 0 µM (blank), 0.001 µM (low dose), or 0.1 µM 1,25(OH)_2_D_3_ (high dose) for 48 h before each examination. *, **, and *** denote *P*<0.05, 0.01, and 0.001 compared with Blank cells, respectively.

MCF-7 cells treated with 1,25(OH)_2_D_3_ showed a dose-dependent increase in the percentage of apoptotic cells and a dose-dependent decrease in proliferation ability. When compared with the percentage of EdU^+^ cells among blank cells (51.9 ± 4.8%), the EdU^+^ percentages were significantly lower among MCF-7 cells treated with 0.001 µM (25.5 ± 4.8%) or 0.1 µM 1,25(OH)_2_D_3_ (7.5 ± 1.5%) (*P*<0.01 and *P*<0.001, respectively). Furthermore, the percentage of cells in G_0_/G_1_ phase was markedly increased and the percentage in G_2_/S phase was dramatically reduced (Figure 1A). In contrast, the percentages of apoptotic cells among cells treated with 0.001 µM (25.3 ± 0.9%) or 0.1 µM 1,25(OH)_2_D_3_ (40.2 ± 1.6%) were significantly higher than the percentage among blank control cells (7.6 ± 0.5%; *P*<0.01 and *P*<0.001, respectively; Figure 1B). Results of the Hoechst 33258 immunofluorescence assay confirmed that 1,25(OH)_2_D_3_ administration had indeed induced more aggregation or brilliant blue spots in cells, indicating that DNA damage had occurred in a dose-dependent manner (Figure 1C). These results suggested that 1,25(OH)_2_D_3_ had suppressed the proliferation ability of the MCR-7 cells and induced cell apoptosis by causing DNA damage.

### Vitamin D administration enhanced VDR expression

Because proper function of the VDR requires the bioactivation of vitamin D, we detected the level of VDR protein expression in response to vitamin D administration in MCF-7 cells. As expected, we detected an up-regulation of VDR protein expression in the MCR-7 cells treated with 1,25(OH)_2_D_3_ (Figure 1D). These results indicated that administration of 1,25(OH)_2_D_3_ to cultured MCF-7 cells indeed activated vitamin D-mediated metabolic processes.

### MCF-7 stem cells showed a relatively higher resistance to tamoxifen

**Figure 2:**
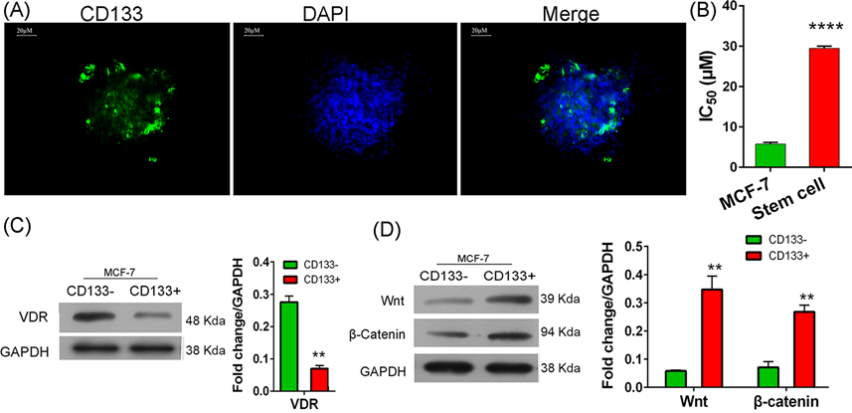
Characteristics of CSCs (**A**) Spheroids identified by an immunofluorescence assay performed with FITC-conjugated CD133 antibodies; scale bar = 20 µm. (**B**) The tamoxifen concentration that reduced the numbers of viable parental MCF-7 cells and CD133^+^ CSCs by 50% (IC_50_). (**C,D**), expression of the VDR protein and Wnt/β-catenin signaling factors in CD133^−^ and CD133^+^ cells derived from MCF-7 cells. Cells were sorted using a fluorescence-activated cell sorting method. ** and **** denote *P*<0.01, and *P*=0.0001 compared with blank cells, respectively.

MCF-7 stem cells were sorted by using the CD133 antibody and fluorescence-activated cell sorting methods. MCF-7 stem cell spheroids were identified by using the FITC-conjugated CD133 antibody in an immunofluorescence assay (Figure 2A). Next, we determined that the IC_50_ value of tamoxifen for MCF-7 CSCs (29.44 ± 0.33 µM) was significantly higher than that for parental MCF-7 cells (5.74 ± 0.22 µM, *P*<0.0001; Figure 2B). These results suggested that the stem cells had a higher resistance to tamoxifen.

### VDR expression inactivated Wnt/β-catenin signaling

We next confirmed the presence of VDR expression, and found that VDR expression in CD133^+^ stem cells was lower than that in CD133^−^ cells (*P*<0.01, Figure 2C). However, the levels of Wnt and β-catenin protein expression in CD133^+^ cells were higher than those in CD133^−^ cells (*P*<0.01, Figure 2D), which was contrary to the trend shown by the VDR. These results suggested that VDR expression blocked the Wnt/β-catenin signaling pathway, and CD133^+^ CSCs had a lower capacity for activating vitamin D-mediated metabolic processes. Additionally, the higher resistance of CD133^+^ cells to tamoxifen might be mediated by the activation of Wnt/β-catenin signaling.

### VDR mediates tamoxifen sensitivity in MCF-7 stem cells via the inactivation of Wnt/β-catenin signaling

**Figure 3:**
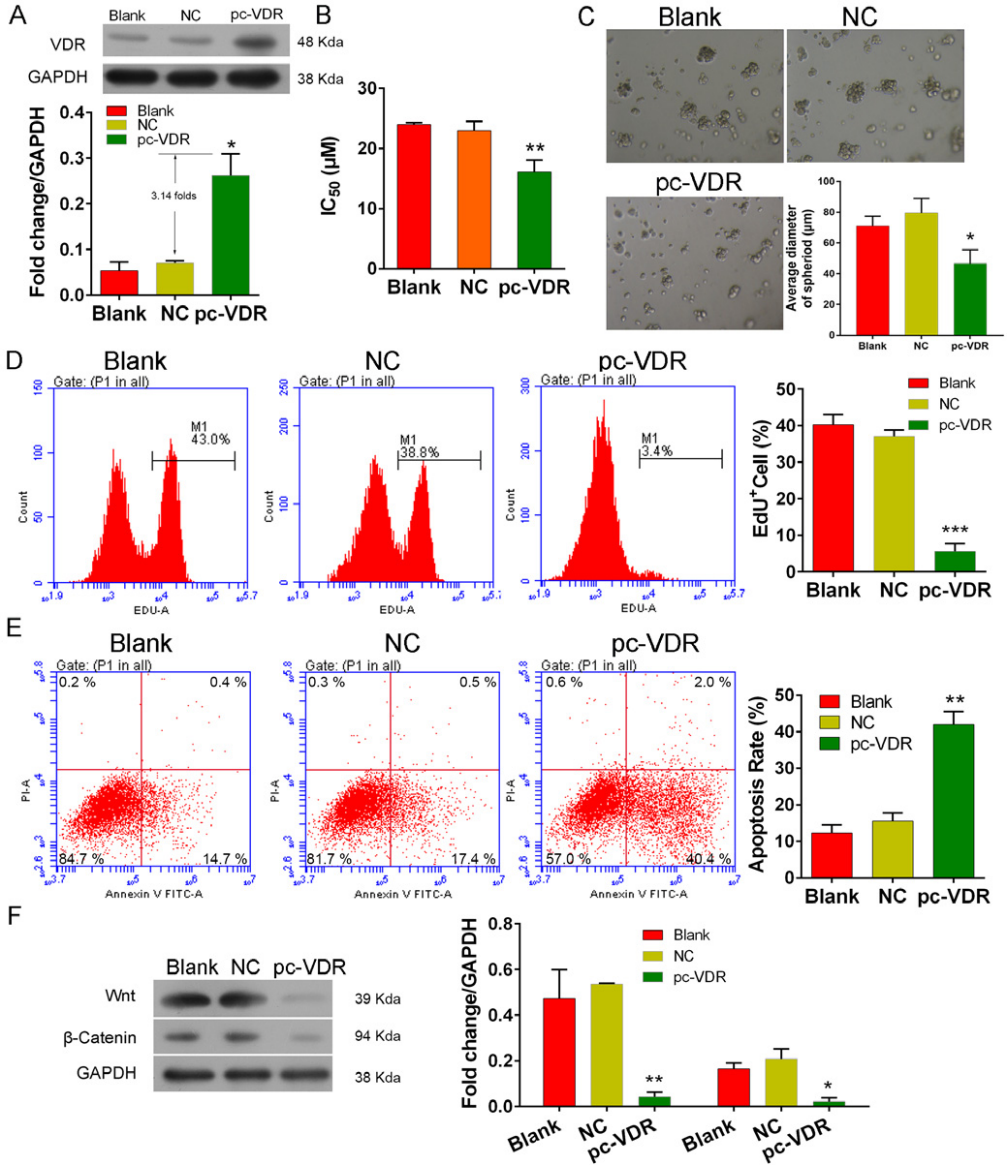
VDR overexpression in CSCs. CSCs were transfected with a VDR overexpression plasmid (pcDNA3.0) and incubated with 0.1 µM 1,25(OH)_2_D_3_ for 7 days or 2 days. (**A**) Expression of the VDR protein in transfected cells. (**B**) The tamoxifen concentration that reduced the numbers of viable transfected CSCs by 50% (IC_50_). (**C**) SEM imaging of CSC spheroids. (**D,E**) EdU and apoptosis flow cytometric analysis, respectively. (**F**) Expression of Wnt/β-catenin signaling factors in VDR-overexpressing CSCs. GAPDH was used as a reference control in the Western blot analyses.*, ** and *** respectively denote P < 0.05, 0.01 and 0.001 compared with NC.

To investigate the association of VDR-mediated tamoxifen sensitivity with activation of Wnt/β-catenin signaling in MCF-7 stem cells, we transfected CSCs with the VDR-overexpression plasmid, pc-VDR. We found up-regulated levels of VDR expression in the transfected cells when compared with VDR expression in the blank cells and negative controls (NC, transfected with pcDNA3.0 vectors, *P*<0.05, Figure 3A). Next, we found that the IC_50_ value of tamoxifen for transfected cells (16.10 ± 1.13 µM) was lower than that for the controls (>22 µM, *P*<0.01, Figure 3B), suggesting that overexpression of the VDR had sensitized the CSCs to tamoxifen.

In addition, we found that VDR-overexpressing CSCs developed fewer and smaller spheroids (Figure 3C), were less viable (Figure 3D, *P*<0.001), and had higher apoptotic rates (Figure 3E, *P*<0.01) than the control cells (blank and NC). These results indicated that VDR expression enhanced CSC apoptosis and suppressed cell proliferation. Finally, we found that overexpression of the VDR inhibited Wnt and β-catenin protein expression in CSCs, suggesting that VDR expression suppresses Wnt/β-catenin signaling in MCF-7 stem cells. Our results also suggest that overexpression of the VDR in MCF-7 stem cells might sensitize stem cells to tamoxifen by promoting cell apoptosis via the inactivation of Wnt/β-catenin signaling.

### A VDR inhibitor reversed the inhibitory effects induced by 1,25(OH)_2_D_3_

**Figure 4:**
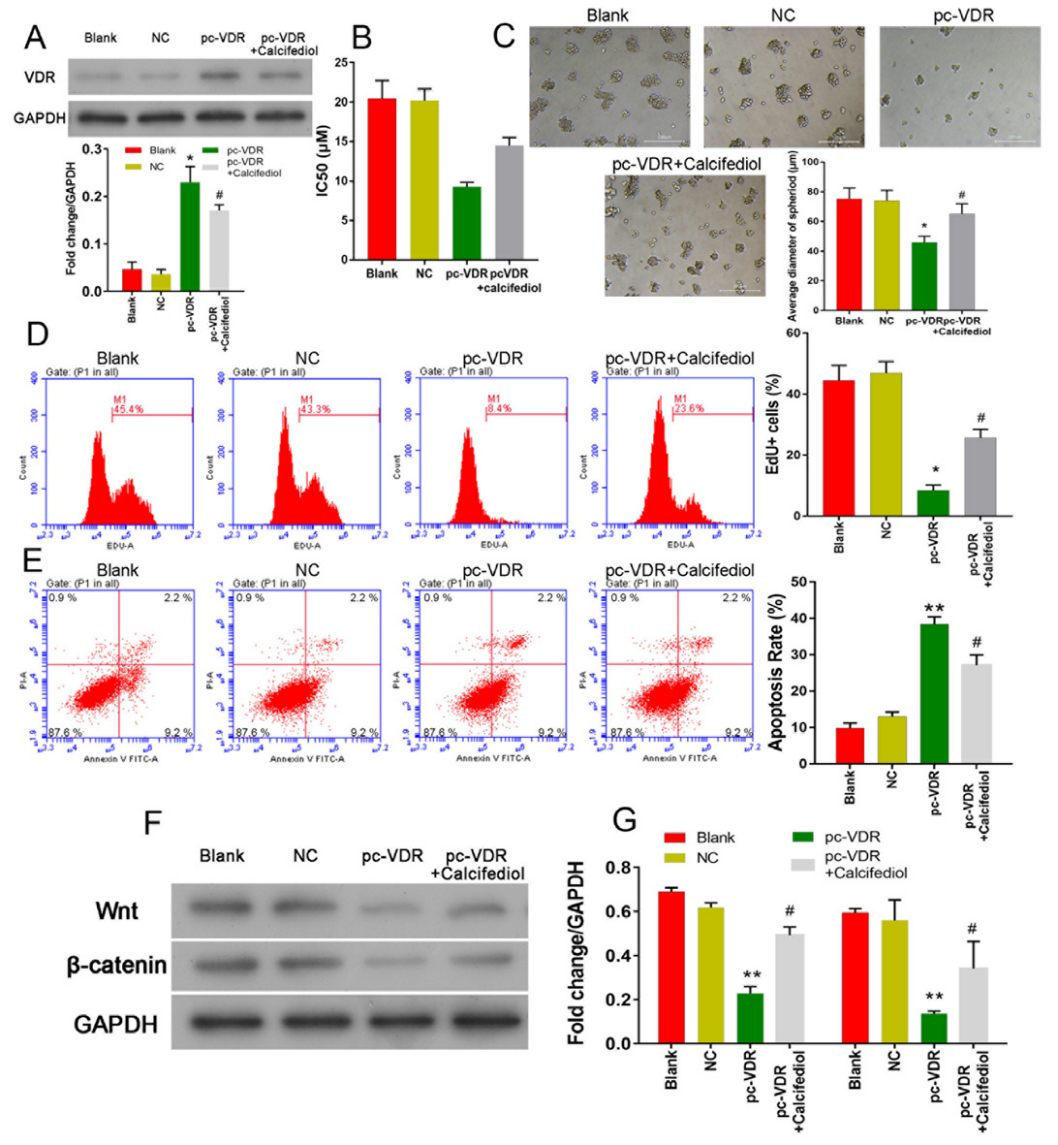
Effect of calcifediol on CSCs overexpressing the VDR. (**A**) Expression of the VDR protein in CSCs was measured by Western blot. (**B**) The IC_50_ value of tamoxifen in each group of CSCs. (**C**) The spheroid formation ability of the CSCs in each group. (**D**) CSC viability was measured by flow cytometry. (**E**) Cell apoptosis rates were measured by flow cytometry. (**F,G**) Expression of Wnt and β-catenin was measured by Western blot methods. *P<0.05 compared with NC; ^#^*P*<0.05 compared with pc-VDR.** denotes P < 0.01 compared with NC.

To further confirm the role of the VD/VDR signaling pathway in breast cancer, the VDR inhibitor, calcifediol was used in the following experiments. First, VDR expression was measured by Western blot methods, and the pcDNA 3.0 VDR was shown to significantly promote VCR expression in stem-like cells. However, a significant suppression of VDR expression was detected in cells that had been treated with calcifediol (*P*<0.05, Figure 4A). CCK8 assay results showed that cells that highly expressed the VDR were also more sensitive to tamoxifen, and calcifediol could attenuate that trend. We also found that cells with higher levels of VDR expression formed relatively smaller spheroid bodies (*P*<0.05, Figure 4C), and this trend could be reversed by treatment with the VDR inhibitor.

Furthermore, EdU staining results showed that cell viability was significantly decreased (*P*<0.05, Figure 4D), and cell apoptosis rates were increased (*P*<0.05, Figure 4E) when the VDR was overexpressed. However, cell survival was promoted by treatment with calcifediol, as shown by higher cell viability rates and lower rates of apoptosis (*P*<0.05, Figure 4D,E). Further experiments showed that overexpression of the VDR promoted Wnt and β-catenin expression, and their expression could be suppressed by calcifediol treatment.

## Discussion

CSCs play crucial roles in the initiation, metastasis, and drug resistance of tumors [6–8]. Many CSCs from malignancies have been identified during the past decades, and been reported to mediate tumor metastasis and chemoradiotherapy resistance [9,10]. In our study, we found that when compared with parental MCF-7 cells, MCF-7 stem cells had a higher IC_50_ value for tamoxifen. Therefore, we became interested in the tamoxifen sensitivity mediated by MCF-7 stem cells.

Previous studies have provided insights into effects of 1,25(OH)_2_D_3_, a VDR agonist, and the VDR, and how their interaction helps to regulate cell differentiation, the cell cycle, autophagy, and apoptosis [1,25,26]. Artaza et al. [26] reported that administration of the VDR ligand 1,25D inhibited the proliferation of mesenchymal multipotent cells by arresting the cell cycle at the G_0_/G_1_ phase, rather than by inducing apoptosis. They also found that the cells could increase in size without an induction of Wnt-1 expression. Hager et al. [27] reported that administration of 1,25(OH)_2_D_3_ to squamous cell head and neck carcinoma cell lines induced G_0_/G_1_ cell cycle arrest by promoting VDR-mediated p21 and p27 expression. Genes regulated by 1,25(OH)_2_D_3_ not only modulate the cell cycle, but are also involved in apoptosis [1]. In our present study, we demonstrated that administration of 1,25(OH)_2_D_3_ to MCF-7 cells promoted G_0_/G_1_ cell cycle arrest, and cell apoptosis by inducing expression of the VDR. These results suggest that 1,25(OH)_2_D_3_-induced VDR expression might be responsible for 1,25(OH)_2_D_3_-mediated MCF-7 cell apoptosis.

Given that CSCs are responsible for the initiation, metastasis, and drug resistance of cancers, and activation of the Wnt signaling pathway in CSCs is required for tumorigenesis and metastasis [5–8,12], we sorted MCF-7 stem cells by using fluorescence-activated cell sorting methods and the CD133 antibody to examine VDR expression and activation of Wnt/β-catenin signaling in MCF-7 cells. We first confirmed that the IC_50_ value of tamoxifen for MCF-7 stem cells was higher than that for the parental MCF-7 cells, suggesting tamoxifen resistance. Next, we found that the levels of VDR expression in CSCs (CD133^+^) were lower than those in the differentiated cells (CD133^−^). As expected, we found that the Wnt/β-catenin signaling pathway was highly activated in MCF-7 stem cells. In contrast, the VDR overexpression-induced inactivation of Wnt/β-catenin signaling produced tamoxifen sensitivity in MCF-7 stem cells. Taken together, these results suggest that the activated Wnt/β-catenin signaling pathway was responsible for the tamoxifen resistance of MCF-7 stem cells.

Persistent activation of the oncogenic Wnt/β-catenin signaling pathway in cancer cells is critical for tumor initiation and metastasis, because it promotes the accumulation of its target genes [5,20]. The 1,25D ligand-activated VDR in colon carcinoma cells competes with a β-catenin binding member (Tcf4) for β-catenin binding [28,29]. Increasing evidence suggests that vitamin D or a 1,25(OH)_2_D_3_ supplement can inhibit tumor growth and inactivate Wnt/β-catenin signaling by promoting the binding of β-catenin to the VDR, thus blocking the transduction of Wnt/β-catenin signaling [30,31]. Increased expression of the VDR or administration of vitamin D and its agonists served to inhibit or negatively regulate Wnt/β-catenin signaling, as well as tumorigenesis and metastasis [29,32,33]. Vitamin D administration also induces the expression of E-cadherin, and thus inhibits EMT progression by modulating the β-catenin/TCF-4 axis, which exerts an opposite effect on the β-catenin signaling pathway, and thereby suppresses tumorigenesis and the metastasis of colon carcinoma cells [28]. In the present study, we demonstrated a negative regulatory relationship between the VDR and Wnt/β-catenin signaling. The administration of 1,25(OH)_2_D_3_ to MCF-7 cells and overexpression of the VDR in MCF-7 stem cells indeed induced VDR expression and inhibited MCF-7 cell proliferation and the expression of Wnt and β-catenin proteins. Accordingly, we suggest that these results demonstrate that administration of vitamin D or 1,25(OH)_2_D_3_ to MCF-7 cells can inhibit cell proliferation and increase cell sensitivity to tamoxifen by suppressing Wnt/β-catenin signaling.

## Conclusion

Taken together, we found that administration of 1,25(OH)_2_D_3_ to MCF-7 cells arrested the cells at their G_0_/G_1_ phase, inhibited cell proliferation, promoted cell apoptosis, and caused DNA damage. Administration of vitamin D activated the VDR, which subsequently inhibited Wnt/β-catenin signaling and thus increased cell sensitivity to tamoxifen. We also demonstrated that the resistance of MCF-7 cells to tamoxifen was mainly mediated by MCF-7 stem cells, and that overexpression of the VDR in stem cells increased tamoxifen sensitivity in MCF-7 stem cells by inhibiting Wnt/β-catenin signaling. Vitamin D supplementation, a stimulation of VDR expression, or the inhibition of Wnt/β-catenin signaling in breast CSCs might be a useful approach for the clinical treatment of breast cancers.

## Abbreviations

BD: Becton Dickinson
bFGF: Basic fibroblast growth factor
CCK8: Cell-counting kit-8
CDS: Coding sequences
CSC: cancer stem cells
DMEM: Dulbecco’s Modified Eagle Medium
ECL: Enhanced chemiluminescence
EGF: Epidermal growth factor
EMT: Epithelial mesenchymal transition
FITC: Fluorescein isothiocyanate
HRP: Horse radish peroxidase
MCF-7: Michigan Cancer Foundation-7
VDR: vitamin D receptor

## References

[1] Narvaez C.J. (2014) The impact of vitamin D in breast cancer: genomics, pathways, metabolism. Front. Physiol. 5, 00213 10.3389/fphys.2014.00213 PMC4055997 24982636

[2] Zheng Y. (2017) Loss of the vitamin D receptor in human breast and prostate cancers strongly induces cell apoptosis through downregulation of Wnt/β-catenin signaling. Bone Res. 5, 17023 10.1038/boneres.2017.23 28944088 PMC5605769

[3] Almendro V. (2014) Genetic and phenotypic diversity in breast tumor metastases. Cancer Res. 74, 1338–1348 10.1158/0008-5472.CAN-13-2357-T 24448237 PMC3963810

[4] Arnold K.M. (2015) Wound healing and cancer stem cells: inflammation as a driver of treatment resistance in breast cancer. Cancer Growth Metastasis 2015, 1–13 10.4137/CGM.S11286 PMC4315129 25674014

[5] Takebe N. (2015) Targeting Notch, Hedgehog, and Wnt pathways in cancer stem cells: clinical update. Nature Rev. Clinical Oncol. 12, 445–464 10.1038/nrclinonc.2015.61 25850553 PMC4520755

[6] Li X. (2017) miR-181b-5p mediates TGF-β1-induced epithelial-to-mesenchymal transition in non-small cell lung cancer stem-like cells derived from lung adenocarcinoma A549 cells. Int. J. Oncol. 51, 158–168 10.3892/ijo.2017.4007 28534939 PMC5467782

[7] Plaks V., Kong N. and Werb Z. (2015) The cancer stem cell niche: how essential is the niche in regulating stemness of tumor cells? Cell Stem Cell 16, 225–238 10.1016/j.stem.2015.02.015 25748930 PMC4355577

[8] Jaggupilli A. and Elkord E. (2012) Significance of CD44 and CD24 as cancer stem cell markers: an enduring ambiguity. Clinical Dev. Immunol. 2012, 708036 10.1155/2012/708036 22693526 PMC3369436

[9] Liu S. (2013) Breast cancer stem cells transition between epithelial and mesenchymal states reflective of their normal counterparts. Stem Cell Reports 2, 78–91 10.1016/j.stemcr.2013.11.009 24511467 PMC3916760

[10] Bartucci M. (2015) TAZ is required for metastatic activity and chemoresistance of breast cancer stem cells. Oncogene 34, 681 10.1038/onc.2014.5 24531710

[11] Todaro M. (2014) CD44v6 is a marker of constitutive and reprogrammed cancer stem cells driving colon cancer metastasis. Cell Stem Cell 14, 342–356 10.1016/j.stem.2014.01.009 24607406

[12] Takebe N. (2011) Targeting cancer stem cells by inhibiting Wnt, Notch, and Hedgehog pathways. Nature Rev. Clinical Oncol. 1, 97–106 10.1038/nrclinonc.2010.196 21151206

[13] Ginestier C. and Wicha M.S. (2007) Mammary stem cell number as a determinate of breast cancer risk. Breast Cancer Res. Bcr. 9, 109 10.1186/bcr1741 17688678 PMC2206714

[14] Gong Y. (2014) Aldehyde dehydrogenase 1 expression in inflammatory breast cancer as measured by immunohistochemical staining. Clin. Breast Cancer 14, e81–e88 10.1016/j.clbc.2013.12.006 24461456

[15] Ferrermayorga G. (2016) Vitamin D receptor expression and associated gene signature in tumour stromal fibroblasts predict clinical outcome in colorectal cancer. Gut 66, 1449 10.1136/gutjnl-2015-310977 27053631 PMC5530491

[16] Bolland M.J. (2014) The effect of vitamin D supplementation on skeletal, vascular, or cancer outcomes: a trial sequential meta-analysis. Lancet Diabetes Endocrinol. 2, 307–320 10.1016/S2213-8587(13)70212-2 24703049

[17] Bolland M.J. (2011) Calcium and vitamin D supplements and health outcomes: a reanalysis of the Women’s Health Initiative (WHI) limited-access data set. Am. J. Clin. Nutr. 94, 1144–1149 10.3945/ajcn.111.015032 21880848 PMC3173029

[18] Feldman D. (2014) The role of vitamin D in reducing cancer risk and progression. Nat. Rev. Cancer 14, 342–357 10.1038/nrc3691 24705652

[19] Bong Y.S. (2016) A role for the vitamin D pathway in non-intestinal lesions in genetic and carcinogen models of colorectal cancer and in familial adenomatous polyposis. Oncotarget 7, 80508–80520 10.18632/oncotarget.12768 27768599 PMC5348337

[20] Arensman M.D. (2015) Abstract B77: Vitamin D suppresses pancreatic cancer growth through inhibition of autocrine Wnt/β-catenin signaling. Cancer Res. 75, [abstract]. In: Proceedings of the AACR Special Conference on Pancreatic Cancer: Innovations in research and Treatment; May 18-21, 2014; New Orleans, LA. Philadelphia (PA):AACR, 10.1158/1538-7445.PANCA2014-B77

[21] So J.Y. and Suh N. (2015) Targeting cancer stem cells in solid tumors by vitamin D. J. Steroid Biochem. Mol. Biol. 148, 79–85 10.1016/j.jsbmb.2014.10.007 25460302 PMC4361233

[22] Ojo D. (2015) Factors promoting tamoxifen resistance in breast cancer via stimulating breast cancer stem cell expansion. Curr. Med. Chem. 22, 2360 10.2174/0929867322666150416095744 25882671

[23] Luo Y. (2015) Single-cell transcriptome analyses reveal signals to activate dormant neural stem cells. Cell 161, 1175–1186 10.1016/j.cell.2015.04.001 26000486 PMC4851109

[24] Boo L. (2016) miRNA transcriptome profiling of spheroid-enriched cells with cancer stem cell properties in human breast MCF-7 cell line. Int. J. Biol. Sci. 12, 427–445 10.7150/ijbs.12777 27019627 PMC4807162

[25] Wu S. (2015) Intestinal epithelial vitamin D receptor deletion leads to defective autophagy in colitis. Gut 64, 1082–1094 10.1136/gutjnl-2014-307436 25080448 PMC4312277

[26] Artaza J.N. (2010) 1,25(OH)2 vitamin D3 inhibits cell proliferation by promoting cell cycle arrest without inducing apoptosis and modifies cell morphology of mesenchymal multipotent cells. J. Steroid Biochem. Mol. Biol. 119, 73–83 10.1016/j.jsbmb.2010.01.001 20064609 PMC2828517

[27] Hager G. (2001) 1,25(OH)2 vitamin D3 induces elevated expression of the cell cycle-regulating genes P21 and P27 in squamous carcinoma cell lines of the head and neck. Acta Otolaryngol. 121, 103–109 10.1080/000164801300006353 11270487

[28] Pálmer H.G. (2001) Vitamin D<sub>3</sub>promotes the differentiation of colon carcinoma cells by the induction of E-cadherin and the inhibition of <strong>β</strong>-catenin signaling. J. Cell Biol. 154, 369–388 10.1083/jcb.200102028 11470825 PMC2150773

[29] He W. (2011) Blockade of Wnt/β-catenin signaling by paricalcitol ameliorates proteinuria and kidney injury. J. Am. Society Nephrol. Jasn 22, 90–103 10.1681/ASN.2009121236 PMC3014038 21030600

[30] Sebio A., Kahn M. and Lenz H.J. (2014) The potential of targeting Wnt/Î²-catenin in colon cancer. Expert Opin. Ther. Targets 18, 611–615 10.1517/14728222.2014.906580 24702624

[31] Li J. (2015) Wnt/β-catenin signaling pathway in skin carcinogenesis and therapy. Biomed Res. Int. 2015, 964842 26078973 10.1155/2015/964842 PMC4452418

[32] Shah S. (2006) The molecular basis of vitamin D receptor and β-Catenin Crossregulation. Mol. Cell 21, 799–809 10.1016/j.molcel.2006.01.037 16543149

[33] Pendás-Franco N. (2008) Vitamin D and Wnt/beta-catenin pathway in colon cancer: role and regulation of DICKKOPF genes. Anticancer Res. 28, 2613–2623 19035286

